# Sustained ameliorating effects and autonomic mechanisms of transcutaneous electrical acustimulation at ST36 in patients with chronic constipation

**DOI:** 10.3389/fnins.2022.1038922

**Published:** 2022-11-21

**Authors:** Jie-Yi Zhou, Jian Wang, Bei-Fang Ning, Ye-Dong Hu, Qi Zhao, Wei Tan, Pei-Mei Shi, Zong-Li Yuan, Xin-Wei Feng, Jiande D. Z. Chen, Wei-Fen Xie

**Affiliations:** ^1^Department of Gastroenterology, Changzheng Hospital, Naval Medical University, Shanghai, China; ^2^Department of Gastroenterology, Shanghai East Hospital, Tongji University School of Medicine, Shanghai, China; ^3^Division of Gastroenterology and Hepatology, University of Michigan, Ann Arbor, MI, United States

**Keywords:** chronic constipation, transcutaneous electrical acustimulation, spontaneous bowel movement, anorectal manometry, autonomic function

## Abstract

**Background and aims:**

The treatment of chronic constipation is still a great challenge in clinical practice. This study aimed to determine the efficacy and sustained effects of transcutaneous electrical acustimulation (TEA) at acupoint ST36 on the treatment of chronic constipation and explore possible underlying mechanisms.

**Methods:**

Forty-four patients with chronic constipation were recruited and randomly assigned to a TEA group or sham-TEA group. A bowel diary was recorded by the patients. The Patient Assessment of Constipation Symptom (PAC-SYM) and the Patient Assessment of Constipation Quality of Life (PAC-QoL) questionnaires were administered during each visit. Anal and rectal functions were evaluated with anorectal manometry. Autonomic functions were assessed by the special analysis of heart rate variability derived from the ECG recording.

**Results:**

Compared with sham-TEA, 2-week TEA treatment significantly increased the number of spontaneous bowel movements (SBMs) (5.64 ± 0.54 vs. 2.82 ± 0.36, *P* < 0.001) and lowered the total scores of PAC-SYM (0.90 ± 0.14 vs. 1.35 ± 0.13, *P* < 0.001) and PAC-QoL (0.89 ± 0.13 vs. 1.32 ± 0.14, *P* < 0.05). TEA improved symptoms, as reflected by a reduction in the straining (*P* < 0.001), the incomplete defecation (*P* < 0.05), the frequency of emergency drug use (*P* < 0.05), the days of abdominal distension (*P* < 0.01) and an increase in intestinal satisfaction (*P* < 0.01). Interestingly, the effects of TEA on the improvement of weekly SBMs sustained four weeks after the cessation of treatment (*P* < 0.001). Anorectal manometry indicated that 2-week treatment of TEA lowered the threshold of first sensation (*P* < 0.05), desire of defecation (*P* < 0.01) and maximum tolerable volume (*P* < 0.001) compared with sham-TEA group. TEA also significantly enhanced vagal activity, reflected by high-frequency band of heart rate variability, compared with sham-TEA (57.86 ± 1.83 vs. 48.51 ± 2.04, *P < 0.01*).

**Conclusion:**

TEA ameliorates constipation with sustained effects, which may be mediated via improvement of rectal sensitivity and enhancement of vagal activity.

**Clinical trial registration:**

[https://clinicaltrials.gov/], identifier [ChiCTR210004267].

## Introduction

Chronic constipation is one of the most common gastrointestinal (GI) disorders, affecting approximately 15% of the global population (Bharucha and Lacy, 2020) and 37.7% of people over 80 years old (Chu et al., 2014), and has a substantial impact on both patients and healthcare systems, resulting in an enormous healthcare resource burden (Camilleri et al., 2017; Shah et al., 2021). Chronic constipation is usually characterized by the decreased spontaneous bowel movements (SBMs, <3 per week), difficulty in defecation, incomplete evacuation, excess straining and other accompanying symptoms (Forootan et al., 2018).

Conventional treatments of chronic constipation mainly include lifestyle modification, medications and other auxiliary techniques. Non-pharmaceutical treatment via lifestyle modification is the most widely recommended therapy for chronic constipation (Lindberg et al., 2011; Tack et al., 2011; Park et al., 2012; Bharucha et al., 2013; Gwee et al., 2013; Drossman and Hasler, 2016). However, a large number of patients still have no choice but to turn to drugs when they fail to relieve constipation by lifestyle adjustment. Yet, medications used to treat chronic constipation are not always satisfactory due to inefficacy or their side effects (Ford and Suares, 2011; Blackett et al., 2018). Some laxatives even worsen constipation when used improperly (Herrle et al., 2004). What’s more, laxatives are not able to alleviate all the symptoms caused by constipation (Tran and Di Palma, 2005; Gordon et al., 2016). Although prucalopride, a novel high-affinity selective 5-hydroxytryptamine 4 receptor agonist, has been approved for the treatment of severe chronic constipation (SCC), especially for those patients who couldn’t obtain an appropriate therapeutic response from laxatives, it may also cause a variety of side effects, such as nausea, diarrhea, abdominal pain, and headache, and does not work for a fair proportion of patients (Liu et al., 2021).

Acupuncture is a therapeutic technique which has been used for a long time to treat various GI diseases. Previous studies have demonstrated a favorable effect of acupuncture on the treatment of chronic constipation (Liu et al., 2016). Currently, electroacupuncture (EA) is more commonly used in clinical practice (Chao and Zhang, 2014). EA uses electric current instead of manual manipulation to stimulate acupoints. A recent multicenter, randomized, controlled trial with 560 participants by Liu et al. showed that 8-week EA was non-inferior to prucalopride in increasing the proportion of participants with ≥ 3 mean weekly complete SBMs. There was no difference in the improvement of gastrointestinal discomforts and quality of life of SCC between EA and prucalopride groups. More interestingly, the effects of EA could be sustained for up to 24 weeks after treatment (Liu et al., 2021). However, EA uses needles and can only be performed by healthcare providers or acupuncturists. Patients have to go to a clinic to receive EA treatment, which results in high cost and low patient adherence. Transcutaneous electrical acustimulation (TEA) is a non-invasive needleless modality that stimulates acupuncture points via surface electrodes instead of acupuncture needles. TEA is easy to learn and can be performed at home by patients themselves (Chen et al., 2017). Several recent studies have preliminarily explored the effect of TEA on the treatment of constipation. Zhang et al. (2014) found that TEA at both the posterior tibial nerve (PTN) and Zusanli (ST36) was effective in relieving constipation via the modulation of autonomic function. Wu et al. (2020) designed a cross-over study to compare the effectiveness of TEA at ST36 and TEA at PTN in treating functional constipation and found that TEA was more potent at ST36 than at PTN in improving constipation and the constipation-related symptoms. Liu et al. (2020) reported the ameliorating effects of TEA at the Neiguan (PC6) and ST36 acupoints combined with adaptive biofeedback training in patients with functional outlet obstruction constipation. However, these studies were all conducted in a single center and lacked comprehensive analysis of constipation symptoms and rectal sensation. In addition, it was unknown whether there was a sustained effect on constipation after termination of TEA treatment.

In this randomized, single-blinded, sham-controlled, multicenter study, we aimed to investigate the efficacy of TEA at ST36 on treating constipation, including improvements in constipation-related symptoms and the quality of life. A 4-week follow-up after treatment termination was conducted to clarify the long-term effect of TEA. Rectal sensation and autonomic function were measured to explore the underlying mechanisms of TEA in patients with chronic constipation.

## Materials and methods

### Study participants

Forty-four patients with chronic constipation were recruited for this study at the Department of Gastroenterology, Changzheng Hospital, and the Department of Gastroenterology, Shanghai East Hospital, from June 2020 to March 2021. The inclusion criteria were as follows: (i) willing to follow the treatment plan; (ii) 18-75 years old (regardless of sex); (iii) diagnosed with chronic constipation. The exclusion criteria included (i) history of malignant tumors; (ii) chronic constipation caused by drugs, such as antidepressants, opioids, and antiepileptic drugs; (iii) severe cardio-cerebrovascular disease; (iv) history of abdominal surgery within 6 months (except for appendectomy, cholecystectomy and cesarean section); (v) cognitive impairment, aphasia, mental disorders or diseases that may affect patient cooperation; (vi) being pregnant, preparing to become pregnant or lactating; (vii) being familiar with the position of acupoints or received this therapy previously; (viii) having dermatosis affecting electrode placement; and (ix) researchers determining that this study’s treatment was not suitable.

### Study design

The study protocol was approved by the Ethics Committee of Changzheng Hospital (*No. 2020SL034*) and conformed to the ethical guidelines of the 1975 Declaration of Helsinki. The trial was registered at www.chictr.org.cn (No. ChiCTR2100042676). The protocol was explained to each patient, and written informed consent was obtained from each participant before the procedure. All authors had access to the study data and reviewed and approved the final manuscript.

This was a randomized, single-blinded, sham-controlled, multicenter study. Eligible patients were randomly assigned to the TEA group or sham-TEA group at a ratio of 1:1 according to a computer-generated random digital table. An appropriate sample size was calculated by G*power analyses based on our preliminary study. One week before the study, all patients were asked to stop using laxatives, suppositories other related drugs and stop receiving enemas that can assist with defecation. *Patients were allowed to continue life modifications they used before joining the study but not recommended for any new life modification measures.* The TEA and sham-TEA treatments were performed 1 h twice a day after breakfast and dinner for 14 days for all patients. After termination of the 2-week treatment, a four-week follow-up was conducted for each participant.

The patients were provided a bowel diary to be completed at home. The diary included the following items: number of bowel movements (BMs), number of SBMs, use of emergency drugs, straining duration defecation, incomplete feeling after defecation, time spent per defecation, occurrence of abdominal distension, and abdominal pain. Additionally, the bowel diary was accompanied by a picture of the Bristol Stool Form Scale (BSFS) to help each patient identify and record fecal traits (Jaruvongvanich et al., 2017). If a patient did not defecate spontaneously for 3 consecutive days or was unable to tolerate the symptoms of constipation during the period of treatment at home, polyethylene glycol electrolyte powder was allowed to be taken orally as an emergency treatment solution (1 packet of agent A + 1 packet of agent B dissolved in 125 ml of water each time), and its usage was recorded. During the 2-week treatment, all the participants were required to record the number of times they strained to defecate, the number of incomplete defecations, the time spent on each defecation, the type of BSFS scale, the frequency of emergency drug use, the score of intestinal satisfaction (recorded by a visual analog scale), the days of abdominal pain, the days of abdominal distension and the treatment time of electrical acustimulation per day. Then, the proportion of straining and the proportion of incomplete defecation from the total number of defecations in each patient per week were calculated.

The patients were required to receive 3 office visits (before treatment, the termination of the 2-week treatment and the termination of the 4-week follow-up) during the whole study. The patients were required to complete questionnaires, including the Patient Assessment of Constipation Symptoms (PAC-SYM) and the Patient Assessment of Constipation Quality of Life (PAC-QoL) at each visit. In addition, electrocardiography (ECG) and anorectal manometry (ARM) were performed in the two groups before and after the two-week treatment.

### Assessment of Patient Assessment of Constipation Symptoms and Patient Assessment of Constipation Quality of Life

The PAC-SYM questionnaire, consisting of 12 questions representing stool symptoms, abdominal symptoms and rectal symptoms, was used to evaluate the severity of constipation-related symptoms (Frank et al., 1999). The PAC-QoL questionnaire was adopted to assess the quality of life of patients with chronic constipation; this questionnaire contains a total of 28 items and comprises 4 subscales for the assessments of physical discomfort, psychosocial distress, worries and concerns, and satisfaction (Marquis et al., 2005). The average scores of each subscale and the total subscales were recorded.

### Needleless transcutaneous electrical acustimulation and sham- transcutaneous electrical acustimulation treatments

ST36 (Zusanli) was chosen as the stimulation acupoint for the TEA treatment via a watch-sized digital stimulator (SNM-FDC01: Ningbo Maida Medical Device, Ningbo, China) as previously reported (Zhang et al., 2021). The parameters were set as follows: train on-time of 2 s and off-time of 3 s, pulse width of 0.5 ms, and pulse frequency of 25 Hz; the pulse amplitude of stimulation was adjusted in a range of 2-10 mA based on patient tolerability and preference (Zhang et al., 2014; Liu et al., 2018; Wu et al., 2020). As a control, sham-TEA was performed using the same stimulation parameters as TEA but with electrodes placed at 10-15 cm below and lateral to ST36 at non-acupoints (Zhang et al., 2018). The recruited patients were blinded to the type of treatment they were assigned.

### Efficacy and safety assessment

The primary study endpoints were the number of weekly SBMs, the improvement in PAC-SYM and the improvement in PAC-QoL. The secondary endpoints were time spent on each defecation, stool characteristics according to the Bristol stool chart, frequency of emergency drug usage, intestinal satisfaction score and time spent on TEA treatment.

Safety assessments consisted of monitoring adverse events and the results of clinical laboratory testing if needed. Severe adverse events were defined as those leading to hospitalization, prolonged hospitalization, disability, impact on work capacity, endangered life, or death.

### Evaluation of anal and rectal function

Anorectal manometry (ARM) is an objective and widely applied clinical procedure in the evaluation of both anal and rectal function. In this study, a high-resolution ARM test was measured by using a water-perfused manometric system (a pressure catheter with outer diameter of 4.2 mm and a rectal balloon connected to the distal end; GAP-36A, Ningbo Maida Medical Device, Inc.) (Wu et al., 2020). The manometric catheter was lubricated and then inserted into the anorectum. After resting for a few minutes, the subject was asked to contract the anus three times and then strain it three times. The anorectal inhibitory reflex was then evaluated by inflating the balloon attached to the tip of the catheter with a hand-held syringe to a volume from 0-50 ml. Finally, a syringe was used to inflate the balloon gradually until reaching the subject’s maximum tolerance (Meunier and Gallavardin, 1993; Diamant et al., 1999); during the ramp distention, the patient was asked to report first sensation, desire to defecate, urge to defecate and maximum tolerance.

### Assessment of autonomic functions

Autonomic functions were evaluated by spectral analysis of heart rate variability (HRV). The procedure was conducted as follows: after a 10-min rest, the patient remained in a supine position, and five surface electrodes were placed at the intersection of the midline of the clavicle and the bilateral second rib and eighth rib, and the intersection of the right sternal edge and the third rib. All these electrodes were connected to an ECG amplifier (ECG-201, Ningbo Maida Medical Device, Inc.). The ECG was recorded for 30 min at baseline and 30 min after the 2-week TEA treatment in a fasting state. A previously validated custom-made software was used to derive an HRV signal from the ECG and perform spectral analysis of the HRV signal (Liu S. et al., 2008). The power in the low-frequency band (0.04 to 0.15 Hz; LF) of the HRV spectrum represented mainly sympathetic activity, while the power in the high-frequency band (0.15 to 0.50 Hz; HF) reflected purely vagal or parasympathetic activity. The LF:HF ratio reflected the balance between sympathetic and parasympathetic activity (Sallam et al., 2007; McNearney et al., 2013).

### Statistical analysis

All data were analyzed by SPSS 26.0 (IBM SPSS, Chicago, IL, USA). The data are presented as means ± standard errors (SEs). *P* < 0.05 was considered statistically significant. Paired Student’s *t* test or Wilcoxon test were used to investigate the differences in parameters before and after the treatment. Independent sample *t* tests or Wilcoxon tests were used to compare the differences between the TEA group and the sham-TEA group. The proportions of participants with ≥ 3 weekly SBMs after treatment between the two groups were compared using χ2 tests.

## Results

### Patient demographics and baseline characteristics

A total of 44 patients with chronic constipation were recruited and randomized into sham-TEA and TEA groups at a ratio of 1:1. There were no patient dropouts of patients during the whole study. The demographics and baseline characteristics of the patients are shown in Table 1. There were no significant differences in demographics or baseline characteristics (including age, sex, BMI, smoking, drinking, blood pressure, history of chronic constipation, and comorbidities) between the two groups.

**TABLE 1:** Baseline characteristics of the patients with chronic constipation.

Variables	TEA (*n* = 22)	Sham-TEA (*n* = 22)	*P* value
Number	22	22	
Age (y)	45.36 ± 3.22	52.59 ± 3.05	0.111
Female (%)	16 (72.7%)	14 (63.6%)	0.517
BMI (kg/m^2^)	22.36 ± 0.50	23.34 ± 0.71	0.268
Ex or current smoker	3 (13.6%)	3 (13.6%)	1.000
Alcohol abuse	2 (9.1%)	3 (13.6%)	1.000
Systolic pressure (mmHg)	124.86 ± 2.76	130.23 ± 3.13	0.206
Diastolic pressure (mmHg)	77.36 ± 2.04	80.82 ± 2.33	0.271
History of chronic constipation			
Duration of constipation (y)	12.52 ± 1.97	13.91 ± 2.06	0.629
Weekly SBMs	1.57 ± 0.17	1.48 ± 0.18	0.713
Intestinal satisfaction	15.45 ± 2.77	13.86 ± 3.42	0.719
Comorbidities (%)	4 (18.2%)	4 (20%)	1.000
Diabetes	4 (20%)	4 (15%)	1.000
Thyroid disease	0 (0%)	1 (5%)	1.000

### Effects of TEA on weekly SBMs of patients with chronic constipation

**FIGURE 1:**
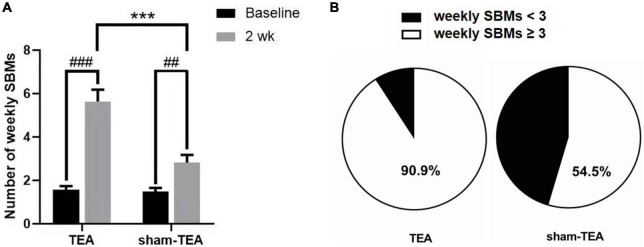
Effects of TEA and sham-TEA on the weekly SBMs of patients with chronic constipation after 2 weeks’ treatment. **(A)** The mean weekly SBMs in the two groups before and after 2 weeks of treatment (vs. baseline, ^##^*P* < 0.01, ^###^*P* < 0.001; vs. sham-TEA, ***P < 0.001). **(B)** The proportion of patients with <3 and ≥3 mean weekly SBMs after 2 weeks of treatment in the two groups.

As shown in Figure 1A, the number of weekly SBMs in patients was significantly increased from 1.57 ± 0.17 at baseline to 5.64 ± 0.54 after 2 weeks of TEA treatment (*P* < 0.001). There was also an improvement in the sham-TEA group (2.82 ± 0.36 vs 1.48 ± 0.18, *P* < 0.01). However, the intergroup analysis showed that the number of weekly SBMs after treatment in the TEA group was significantly higher than that of the sham-TEA group (5.64 ± 0.54 vs. 2.82 ± 0.36, *P* < 0.001). The proportion of participants with ≥3 weekly SBMs after treatment in the TEA group was higher than that in the sham-TEA group (90.9 vs. 54.5%, *P* < 0.05; Figure 1B). These results demonstrated that TEA treatment was able to improve the number of weekly SBMs and was more effective than the sham-TEA treatment.

### Effects of transcutaneous electrical acustimulation on constipation-related symptoms and quality of life

**FIGURE 2:**
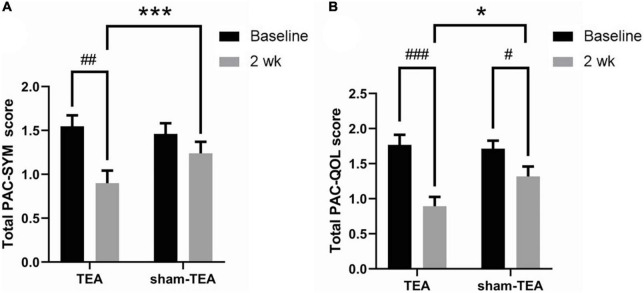
Effects of TEA on the PAC-SYM and the PAC-QoL. The total PAC-SYM score **(A)** and total score of PAC-QoL **(B)** in TEA group and sham-TEA group after 2 weeks of treatment (vs baseline, *^#^P* < 0.05, ^##^*P* < 0.01, ^###^*P* < 0.001; vs sham-TEA, *P < 0.05, ***P < 0.001).

To further explore the efficacy of TEA on the objective symptoms and the quality of life of chronic constipation patients, we collected PAC-SYM and PAC-QoL scale data before and after the treatment. As shown in Figure 2A, the total PAC-SYM score in the TEA group was significantly decreased after 2 weeks of treatment when compared with baseline (0.90 ± 0.14 vs. 1.54 ± 0.13, *P* < 0.01), while there was no significant improvement in the sham-TEA group (1.35 ± 0.13 vs. 1.46 ± 0.12, *P* = 0.538). The total PAC-SYM score in the TEA group was also lower than that of the sham-TEA group after treatment (0.90 ± 0.14 vs. 1.35 ± 0.13, *P* < 0.001). Further analysis of the scores from the three subscales of the PAC-SYM scale showed that the score of stool symptoms, score of abdominal symptoms and score of rectal symptoms after treatment were significantly lower than those before treatment in the TEA group when compared with baseline (all *P* < 0.001, Supplementary Figure 1). Moreover, there was no significant differences in the three subscales in the sham-TEA control group before and after treatment (all *P* > 0.05, Supplementary Figure 1). There was also a significant difference in the score of stool symptoms between the two groups after treatment (*P* < 0.05, Supplementary Figure 1A).

After 2 weeks of TEA treatment, the total PAC-QoL score in the TEA group significantly decreased when compared with baseline (0.89 ± 0.13 vs. 1.77 ± 0.14, *P* < 0.001; Figure 2B). The score in the sham-TEA group was decreased but to a significantly lesser degree than that of the TEA group (1.32 ± 0.14 vs. 1.71 ± 0.12, *P* < 0.05; Figure 2B). The total PAC-QoL score in the TEA group was also lower than that of the sham-TEA group after treatment (0.89 ± 0.13 vs. 1.32 ± 0.14, *P* < 0.05; Figure 2B). The four subscales of the PAC-QoL scale in the two groups were further analyzed. The results showed that TEA treatment for 2 weeks significantly decreased the score of physical discomfort symptoms and the score of psychosocial symptoms when compared with its baseline (*P* < 0.001 and *P* < 0.01, respectively; Supplementary Figures 2A,B). No significant difference in these two scores was observed in the sham-TEA group before and after treatment (*P* = 0.087 and *P* = 0.208, respectively; Supplementary Figures 2A,B). Interestingly, both TEA and sham-TEA treatment showed amelioration effects on the score of worries and concerns (*P* < 0.01 and *P* < 0.05, respectively; Supplementary Figure 2C) and score of satisfaction (*P* < 0.001 and *P* < 0.05, respectively; Supplementary Figure 2D) after treatment compared with their baseline. There were also significantly differences in the score of physical discomfort and the score of satisfaction between the two groups after treatment (all *P* < 0.01, Supplementary Figures 2A,D).

### Effects of transcutaneous electrical acustimulation on bowel diary parameters

Patients with chronic constipation often suffer from various symptoms, such as straining, feeling of incomplete defecation, abdominal pain and abdominal distension. As shown in Supplementary Table 1, the proportion of straining and incomplete defecation was significantly lower in the TEA group than in the sham-TEA group after 2 weeks of treatment (*P* < 0.001 and *P* < 0.05, respectively). In addition, TEA significantly reduced the frequency of emergency drug use compared with that of the sham-TEA control group (0.82 ± 0.31 vs. 1.98 ± 0.40, *P* < 0.05). The intestinal satisfaction after treatment in the TEA group was significantly higher than that of the sham-TEA control group (57.27 ± 4.47 vs. 31.36 ± 5.59, *P* < 0.01). Similarly, the number of days of abdominal distension in the TEA group was significantly lower than that of the sham-TEA group (2.95 ± 0.40 vs. 5.86 ± 0.69, *P* < 0.01). There was no significant difference in defecation time, the number of days of abdominal pain, the scores of the BSFS scale or electrical stimulation duration between the two groups after treatment (all *P* > 0.05).

### Sustained effects of transcutaneous electrical acustimulation on constipation

**FIGURE 3:**
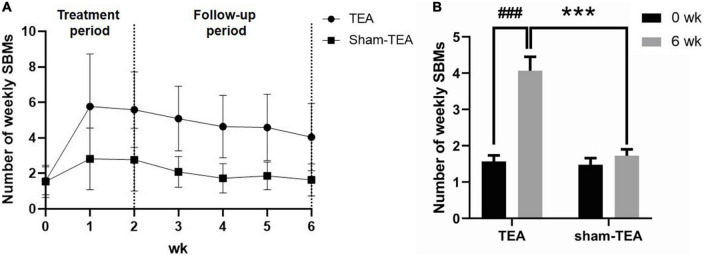
The sustained effect of TEA on the improvement of patients’ weekly SBMs. Weekly SBMs in the TEA group and sham-TEA group during the study **(A)**. Weekly SBMs in the TEA group and sham-TEA group at 6 weeks after treatment **(B)** (vs baseline, ^###^*P* < 0.001; vs sham-TEA, ***P < 0.001).

To explore whether the effects of TEA on chronic constipation were sustained after termination of treatment, we conducted a four-week follow-up after the cessation of the 2-week treatment. The weekly SBMs of the patients in the TEA group and sham-TEA group were recorded every week. In the TEA group, the mean of weekly SBMs increased quickly and peaked ≥5 at week 1 and remained above 4 during the 4-week follow-up period. However, the sham-TEA treatment increased the patients’ weekly SBMs only for the two weeks, reached ≥2 at week 1-2, and decreased gradually to baseline level during the 4-week follow-up period (Figure 3A). The changes from baseline in the TEA group during week 1-6 was greater than that of the sham-TEA group (all *P* < 0.05; Supplementary Table 2). At the end of the follow-up period, the number of weekly SBMs was still notably higher than that at baseline in the TEA group (4.07 ± 0.40 vs. 1.57 ± 0.17, *P* < 0.001), whereas no significant difference was found in the sham-TEA group between baseline and the end of follow-up (1.48 ± 0.18 vs. 1.73 ± 0.18, *P* = 0.086) (Figure 3B). The number of weekly SBMs in the TEA group was significantly higher than that of the sham-TEA group at the same time point (4.07 ± 0.40 vs. 1.73 ± 0.18, *P* < 0.001; Figure 3B). These data suggest that TEA had a sustained effect on the improvement of patients’ weekly SBMs.

### Mechanisms of transcutaneous electrical acustimulation involved in improving rectal sensitivity

To explore the potential mechanisms of TEA involved in improving constipation, we analyzed anal and rectal sensory functions before and after the 2-week treatment. As shown in Table 2, compared with baseline, the threshold of first sensation (55.91 ± 6.02 ml vs. 44.55 ± 4.04 ml, *P* < 0.05), desire for defecation (92.27 ± 6.96 ml vs. 72.72 ± 5.74 ml, *P* < 0.01), urge for defecation (138.18 ± 10.62 ml vs. 113.64 ± 7.10 ml, *P* < 0.01), and maximum tolerable volume (177.73 ± 11.47 ml vs. 145.91 ± 7.97 ml, *P* < 0.001) in the TEA group after treatment all showed a significant decrease, which was not the case in the sham-TEA group (all *P* > 0.05). However, no significant changes were found in rectal resting pressure, anal maximum squeeze pressure or rectoanal inhibitory reflex before and after treatment in either group (all *P* > 0.05). Inter-group comparisons showed that the threshold of first sensation, desire for defecation and maximum tolerable volume in the TEA group were significantly decreased compared to those in the sham-TEA group after treatment (all *P* < 0.05). These results indicate that the improvement of rectal sensitivity was involved in the mechanisms mediating TEA’s therapeutic effect on chronic constipation.

**TABLE 2:** Comparison of parameters of ARM before and after treatment in TEA group (*n* = 22) and sham-TEA group (*n* = 22).

Parameters of ARM	Baseline	TEA	*P* ^#^	Baseline	Sham-TEA	*P* ^¶^	*P* *
Rectal resting pressure	87.00 ± 4.50	84.38 ± 3.46	0.284	84.45 ± 3.78	83.65 ± 2.63	0.788	0.867
Anal maximum squeeze pressure	201.81 ± 13.48	190.16 ± 12.94	0.168	175.54 ± 8.51	166.13 ± 7.80	0.131	0.119
Rectal sensory threshold							
First sensation	55.91 ± 6.02	44.55 ± 4.04	0.014	55.91 ± 5.80	58.64 ± 5.52	0.486	0.046
Desire of defecation	92.27 ± 6.96	72.72 ± 5.74	0.001	90.00 ± 7.67	95.45 ± 7.67	0.174	0.022
Urge of defecation	138.18 ± 10.62	113.64 ± 7.10	0.005	125.91 ± 7.75	132.73 ± 7.03	0.096	0.063
Maximum tolerable volume	177.73 ± 11.47	145.91 ± 7.97	<0.001	164.09 ± 8.26	169.09 ± 7.89	0.316	0.045
Rectoanal inhibitory reflex	18.18 ± 1.07	17.73 ± 1.13	0.329	19.55 ± 1.54	20.00 ± 1.47	0.576	0.228

Rectal resting pressure and anal maximum squeeze pressure are in mmHg; Rectal sensory threshold and rectoanal inhibitory reflex are in ml. *P*^#^: TEA vs baseline, *P*^¶^: sham-TEA vs. baseline, P*: TEA vs sham-TEA.

### Autonomic mechanisms underlying the therapeutic effects of transcutaneous electrical acustimulation on chronic constipation

**FIGURE 4:**
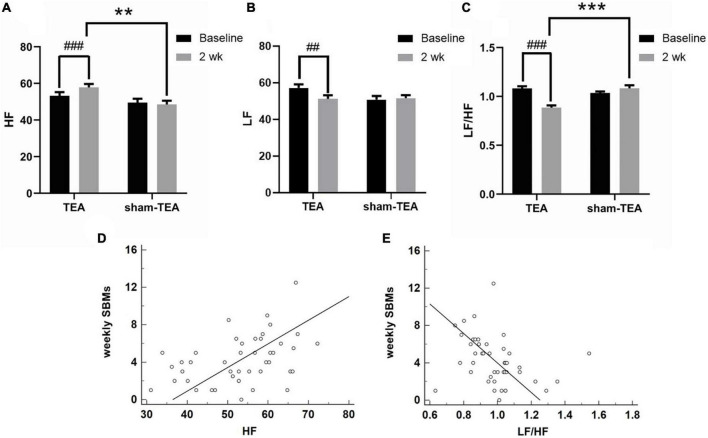
Effects of TEA on autonomic function. The HF **(A)**, LF **(B)** and LF/HF ratio **(C)** in TEA group and sham-TEA group after 2 weeks of treatment (vs. baseline, ^##^*P* < 0.01, ^###^*P* < 0.001; vs. sham-TEA, **P < 0.01, ***P < 0.001). The correlation between weekly SBMs and HF **(D)**. The correlation between weekly SBMs and the LF/HF ratio **(E)**.

Autonomic dysfunction is an important pathogenesis of chronic constipation (Mazur et al., 2012; Jang et al., 2017). Therefore, we derived HRV signals from ECG recordings and analyzed their frequency spectrum to explore the effects of TEA on autonomic function in patients with chronic constipation. As shown in Figure 4A, the HF value assessed from HRV increased from 53.26 ± 1.95 at baseline to 57.86 ± 1.83 at the end of the 2-week treatment in the TEA group (*P* < 0.001), whereas there was no significant change in the HF value between baseline and after treatment in the sham-TEA group (49.50 ± 2.15 vs. 48.51 ± 2.04, *P* = 0.600). In contrast, the LF value decreased from 57.26 ± 1.92 at baseline to 51.21 ± 2.06 after treatment in the TEA group (*P* < 0.01). Similarly, there was no significant difference in the LF value between baseline and after treatment in the sham-TEA group (50.87 ± 1.95 vs. 51.64 ± 1.64, *P* = 0.693; Figure 4B). The ratio of LF/HF was significantly lower than that at baseline in the TEA group after 2-week treatment (0.89 ± 0.02 vs. 1.08 ± 0.02, *P* < 0.001), while the sham-TEA treatment did not affect the LF/HF ratio (1.08 ± 0.03 vs. 1.03 ± 0.01, *P* = 0.123) (Figure 4C). There were significant differences in HF (57.86 ± 1.83 vs. 48.51 ± 2.04, *P* < 0.01) and LF/HF ratio (0.89 ± 0.02 vs. 1.08 ± 0.03, *P* < 0.001) between the two groups after treatment. Further analysis demonstrated that the number of weekly SBMs was positively correlated with HF (*r* = 0.412, *P* < 0.01; Figure 4D) and negatively correlated with LF/HF (*r* = −0.317, *P* < 0.05; Figure 4E). Together, TEA significantly increased vagal activity and decreased sympathetic activity in patients with chronic constipation. These data suggested that the modulation of autonomic function might contribute to the therapeutic effects of TEA on chronic constipation.

## Discussion

The high prevalence of chronic constipation and the symptoms caused by this disease severely affects patients’ quality of life. In this prospective, multicenter, randomized controlled clinical study, we found that TEA substantially increased the number of weekly SBMs, alleviated constipation-related symptoms and improved the quality of life in patients with chronic constipation. Through a 4-week follow-up period after cessation of treatment, we also observed a sustained effect of TEA on constipation, which was not obtained by sham-TEA treatment. The ARM measurement showed that TEA significantly improved rectal sensitivity when compared with sham-TEA after 2 weeks of treatment. TEA also enhanced vagus nerve activity and restore the balance of autonomic function in the chronic constipation patients.

Originating from traditional Chinese medicine, EA was reported to be effective in treating constipation in a previous multi-center study with a cohort of 1075 patients experiencing chronic severe functional constipation (Liu et al., 2016) and was non-inferior to prucalopride in 560 patients with SCC (Liu et al., 2021). In the present study, we demonstrated that single acupoint stimulation at ST36 by needleless TEA not only substantially increased weekly SBMs but also improved constipation-related symptoms and patients’ life quality. More interestingly, we found that the effect of TEA on chronic constipation lasted at least 4 weeks after termination of treatment, which was concordant with the EA results reported by Liu et al. Based on these results, we concluded that needleless and self-administrable TEA could be used as an easy-to-implement and low-cost modality for patients with chronic constipation.

From previous studies, we can find that the placebo effect is commonly observed in patients with functional gastrointestinal diseases when using medications or acupuncture (Zhang et al., 2014; Morishita et al., 2021; Wang et al., 2022). Recently published research demonstrates that acupuncture therapy not only has specific therapeutic effects, but also causes non-specific effects influenced by the placebo effect, expectancy effect, Hawthorne effect and Pygmalion effect (Gong et al., 2019). Herein, we also found that two weeks of sham-TEA treatment increased the number of weekly SBMs from 1.48 ± 0.18 to 2.82 ± 0.36 in chronic constipation patients. However, the improvement in weekly SBMs was more obvious in the TEA group than in the sham-TEA group. Importantly, there were significant differences in the mean weekly SBMs at the end of treatment and the end of the follow-up between the two groups. During the follow-up period, we also found that the non-specific effects associated with sham-TEA quickly disappeared since the stimulation was stopped. These data suggest the therapeutic effect of TEA on chronic constipation is mainly based on the specific effect of acupuncture.

Previous studies have reported that impairment of rectal sensitivity occurs in patients with constipation (Wu et al., 2020; Vollebregt et al., 2021). Rectal sensory thresholds were reported to be increased in constipated patients (Liu T.T. et al., 2008). Zhang et al. reported that transcutaneous neuromodulation at posterior tibial nerve and ST36 significantly decreased the threshold volume to elicit rectoanal inhibitory reflex, ameliorated rectal sensory threshold and maximum tolerance in patients with chronic constipation (Zhang et al., 2014). They later found that transcutaneous neuromodulation at ST36 was able to decrease the urge threshold to rectal distention and the maximum tolerance threshold (Wu et al., 2020). In the current study, TEA at single acupoint ST36 was found to ameliorate rectal sensation by reducing the threshold of first sensation, desire for defecation and maximum tolerable volume. Taken together, these data suggest rectal sensitivity modulation is involved in the therapeutic effect of TEA on chronic constipation.

The autonomic nervous system is known to play a key role in gastrointestinal motility, and the parasympathetic nerve interacts with the enteric nervous system, which is crucial for normal rectal sensation (Wu et al., 2020). In a canine study, electrical stimulation at ST36 restored rectal distention-induced impairment in colonic motility by enhancing vagal activity (assessed by spectral analysis of HRV) mediated via the cholinergic pathway (Jin et al., 2015). In this study, TEA at ST36 was performed using the same parameters as in the previous canine study and enhanced vagal activity was assessed by the same method. It was further demonstrated that the number of weekly SBMs of patients was positively correlated with HF and negatively correlated with LF/HF. These agreements suggest that the improvement in constipation with TEA was at least partly attributed to enhancement in vagal activity.

There were some limitations in our study. (i) Our study was single-blinded, and the patients completed TEA/sham-TEA at home. Although patients familiar with the position of acupoints were excluded, the information bias caused by patients’ subsequent understanding of acupoint distribution was hard to avoid. (ii) Although the study was conducted in two centers, we did not employ a large sample size in this study, which might cause bias in statistical analysis. (iii) We only conducted a 4-week follow-up after treatment. Thus, it is still uncertain the exact effective duration of this technique. *However, a recent study in patients with constipation dominant irritable bowel syndrome using a similar TEA method, demonstrated a sustained effect on constipation 5 months after a 4-week treatment* (Huang et al., 2022).

In conclusion, needleless TEA at single acupoint ST36 alleviates constipation and related symptoms as well as improves the quality of life in patients with chronic constipation, and the effects are sustained for at least 4 weeks. The mechanism of the therapeutic effect of TEA on chronic constipation may be related to improvements in rectal sensitivity and enhancement of parasympathetic activity. Studies with longer follow-up periods and larger sample sizes are necessary to fully determine the potential of this treatment in the future.

## Data availability statement

The raw data supporting the conclusions of this article will be made available by the authors, without undue reservation.

## Ethics statement

The studies involving human participants were reviewed and approved by Ethics Committee of Changzheng Hospital (Shanghai, China). The patients/participants provided their written informed consent to participate in this study.

## Author contributions

J-YZ, JW, and W-FX designed the research and drafted the manuscript. B-FN and Y-DH presided over the enrollment and exclusion of patients. QZ, WT, P-MS, Z-LY, and W-FX followed up the patients and collected the data. B-FN checked the data. J-YZ and JW statistically analyzed the data. JC supervised the conduction of the study and revised the manuscript. All authors contributed to the article and approved the submitted version.

## Supplementary material

Supplementary Figure 1 Effects of TEA on the subscales of the PAC-SYM. The score of stool symptoms **(A)**, score of abdominal symptoms **(B)** and score of rectal symptoms **(C)** in TEA group and sham-TEA group after 2-week treatment (vs. baseline, ^###^*P* < 0.001; vs. sham-TEA, **P* < 0.05).

Supplementary Figure 2 Effects of TEA on the subscales of the PAC-QoL. The score of physical discomfort **(A)**, score of psychosocial domains **(B)**, score of worries and concerns **(C)** and score of satisfaction **(D)** in TEA group and sham-TEA group after 2-week treatment (vs. baseline, ^##^*P* < 0.01, ^###^*P* < 0.001; vs. sham-TEA, ^**^*P* < 0.01).
