# Supplementary material for: Sustained ameliorating effects and autonomic mechanisms of transcutaneous electrical acustimulation at ST36 in patients with chronic constipation
Source: Front Neurosci. 2022 Nov 21;16:1038922. doi: 10.3389/fnins.2022.1038922 (PMC9720110; doi:10.3389/fnins.2022.1038922)
Supplement: Supplementary file 1 [file Data_Sheet_1.docx]

**Supplement Table 1. Bowel diary parameters after 2 weeks’ treatment in TEA group and sham-TEA group.**

|  | TEA (n = 22) | Sham-TEA (n = 22) | *P* |
| --- | --- | --- | --- |
| Proportion of straining | 0.53 ± 0.06 | 0.82 ± 0.05 | 0.000 |
| Proportion of incomplete defecation | 0.38 ± 0.06 | 0.61 ± 0.08 | 0.026 |
| The frequency of emergency drug use | 0.82 ± 0.31 | 1.98 ± 0.40 | 0.025 |
| Time spent on each defecation | 6.77 ± 0.78 | 9.78 ± 1.39 | 0.066 |
| Intestinal satisfaction | 57.27 ± 4.47 | 31.36 ± 5.59 | 0.001 |
| Days of abdominal pain | 0.77 ± 0.25 | 1.64 ± 0.36 | 0.058 |
| Days of abdominal distension | 2.95 ± 0.40 | 5.86 ± 0.69 | 0.001 |
| Type of BSFS scale | 3.945 ± 0.34 | 2.55 ± 0.31 | 0.055 |
| Time of using TEA/sham-TEA | 1.94 ± 0.05 | 1.82 ± 0.08 | 0.191 |

**Supplement Table 2.** The changes of weekly SBMs from baseline in TEA and sham-TEA group in week 1-6 during the study.

| Time | sham-TEA group (n = 22) | TEA (n = 22) | *P* |
| --- | --- | --- | --- |
| Week 1-basline  Week 2-basline  Week 3-basline  Week 4-basline  Week 5-basline  Week 6-basline | 1.27 ± 1.74  1.23 ± 1.56  0.55 ± 0.78  0.18 ± 0.57  0.32 ± 0.82  0.09 ± 0.73 | 4.18 ± 2.90  4.00 ± 1.91  3.50 ± 1.59  3.05 ± 1.58  3.00 ± 1.60  2.45 ± 1.62 | 0.0003  < 0.0001  < 0.0001  < 0.0001  < 0.0001  < 0.0001 |
