# Supplementary figures and images for: Sustained ameliorating effects and autonomic mechanisms of transcutaneous electrical acustimulation at ST36 in patients with chronic constipation
Source: Front Neurosci. 2022 Nov 21;16:1038922. doi: 10.3389/fnins.2022.1038922 (PMC9720110; doi:10.3389/fnins.2022.1038922)

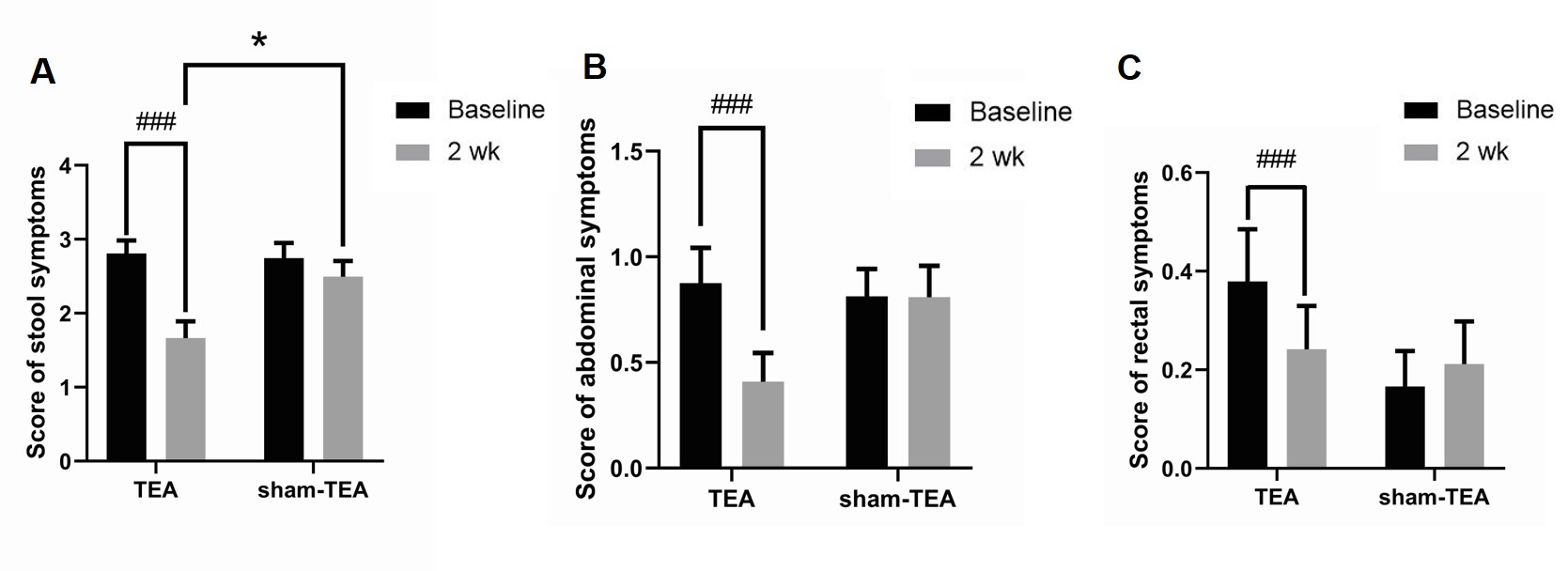

Supplement: Supplementary Figure 1 — Effects of TEA on the subscales of the PAC-SYM. The score of stool symptoms (A), score of abdominal symptoms (B) and score of rectal symptoms (C) in TEA group and sham-TEA group after 2-week treatment (vs. baseline, ###P < 0.001; vs. sham-TEA, *P < 0.05). [file Image_1.JPEG]

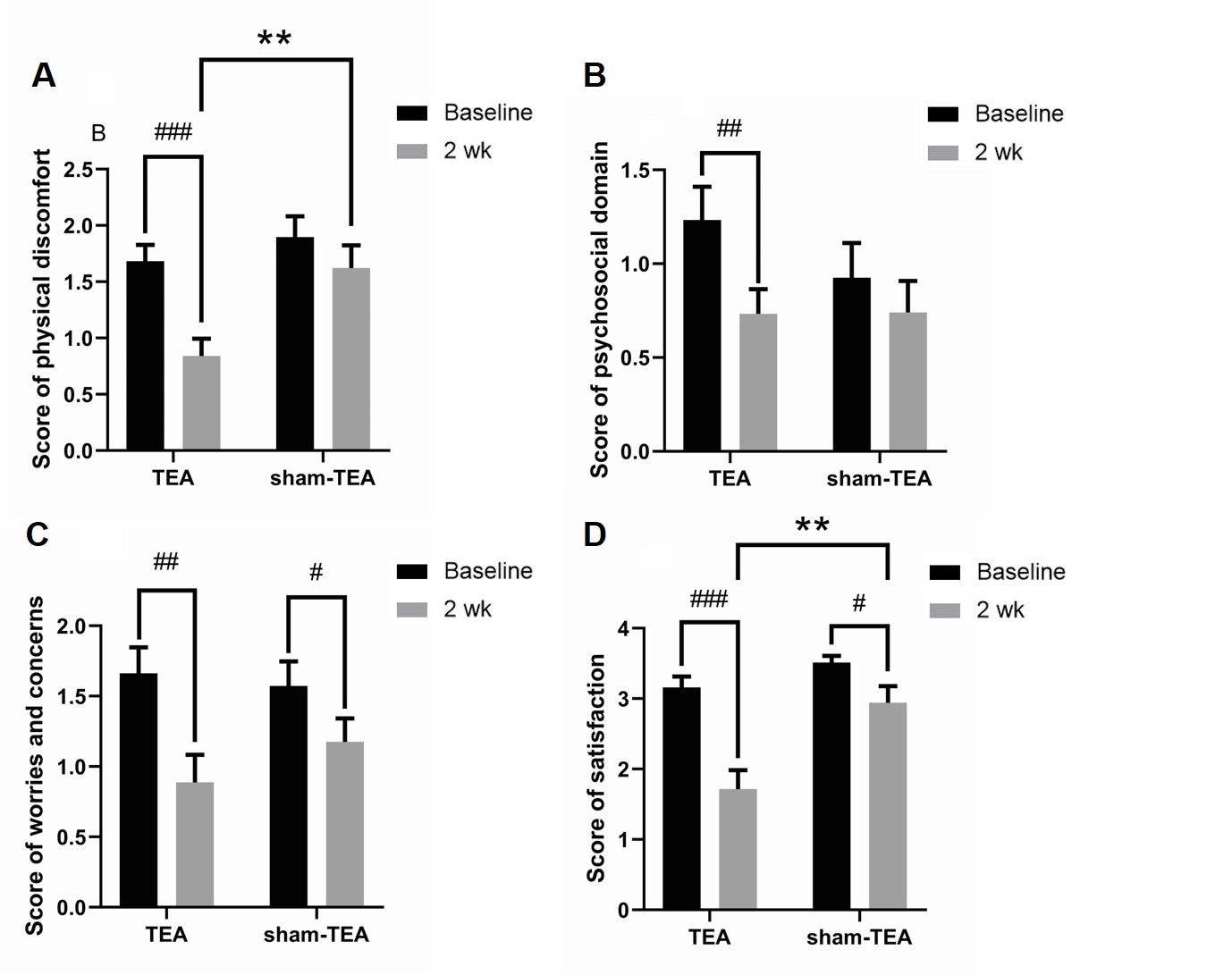

Supplement: Supplementary Figure 2 — Effects of TEA on the subscales of the PAC-QoL. The score of physical discomfort (A), score of psychosocial domains (B), score of worries and concerns (C) and score of satisfaction (D) in TEA group and sham-TEA group after 2-week treatment (vs. baseline, ##P < 0.01, ###P < 0.001; vs. sham-TEA, **P < 0.01). [file Image_2.JPEG]
